# Teaching Dental Drawings for Freshman Dental Students and Its Correlation with Manual Dexterity

**DOI:** 10.1155/2023/5685003

**Published:** 2023-06-01

**Authors:** Hanan Elgendy, Xiaoxi Cui, Todd Watkins, Michelle McQuistan

**Affiliations:** ^1^Operative Division, Department of General Dentistry, School of Dental Medicine, East Carolina University, Greenville, NC, USA; ^2^Department of General Dentistry, School of Dental Medicine, East Carolina University, Greenville, NC, USA; ^3^Department of Preventive & Community Dentistry, University of Iowa College of Dentistry and Dental Clinics, Iowa City, IA, USA

## Abstract

**Objective:**

To assess whether the implementation of teeth drawing exercises in a dental anatomy course improves first-year (D1) dental students' understanding of tooth morphology, their dexterity, and their clinical skills compared with D1s who did not participate in the drawing exercises.

**Methods:**

In 2020, a “Teeth Drawing Module” was implemented within the D1 dental anatomy curriculum. In this course, students learn how to draw accurate outlines of teeth. The students are required to complete two types of drawing projects. Illustrations and instructions of teeth drawings that are outlined in a manual drawing book, PowerPoint presentations, illustration videos, and assessments are provided. Students' grades in the drawing module, waxing skills assessments, and their didactic exams were used to evaluate and assess the correlation between their drawing aptitude and their manual skills. Students who took the drawing course were compared with students who did not take the drawing course to determine if the drawings improved students' understanding of tooth morphology, their dexterity, and their clinical skills. A comprehensive survey was also developed and distributed to students who had the drawing module in their curriculum.

**Results:**

Students who participated in the drawing module were more successful in the dental anatomy course compared with students in the control classes. Classes that had drawing exercises scored significantly higher in all dental anatomy waxing exercises compared with classes that did not have drawing exercises (*p* < 0.001). There was a significant positive correlation between drawing and waxing scores (*p* < 0.05). Moreover, there was a significant positive correlation between drawing and didactic scores (*p* < 0.001).

**Conclusions:**

Drawing exercises can be useful instruments for effectively representing and integrating the spatial domain of anatomical information. Teeth drawings as an adjunctive tool offer excellent visualization and allow students to improve their manual dexterity and knowledge in the dental anatomy course.

## 1. Introduction

It is well established that learning theory and techniques in dentistry can be challenging for predoctoral dental students [1]. Students are required to gain essential knowledge and develop related practical skills in a relatively short period. Specifically, they need to integrate their didactic knowledge with their clinical motor skills and show performance improvement to achieve the competencies required to provide patient care.

Manual dexterity is the ability to use both hands skillfully in a coordinated way to grasp and manipulate objects and demonstrate small precise movements. Dentistry is a medical profession that requires fine motor skills [2], hand–eye coordination [3], and spatial perception [4].

The main goals of restorative dentistry are to restore the form, function, and esthetics of damaged teeth, so understanding the anatomy of anterior and posterior teeth is crucial. As a foundational course in the preclinical dental curriculum, dental anatomy introduces students to the anatomical and morphological characteristics of human permanent and primary dentition.

The course aims to provide first-year (D1) dental students with basic knowledge of dental anatomy and dental terminology to be able to communicate properly with faculty and peers and to apply these concepts during other areas of the dental career. This course also helps the student to develop the psychomotor skills necessary to proficiently reproduce tooth contours and anatomy in wax to be able to apply these skills in their future preclinical and clinical work.

Most predoctoral dental programs aim to teach students basic shapes and predictable patterns so that they can recognize individual teeth and compare them with each other. The anatomical complexity and variation of different teeth can make dental anatomy a difficult subject to learn.

One of the main limitations of the dental anatomy course is that it is usually isolated from other courses related to patient care. Moreover, the traditional lecture model presents several limitations, including one-way communication, lack of interaction, and poor student engagement. Studying the theory of dental anatomy alone is not enough to help the students learn the anatomy of each tooth in detail. Although using the waxing technique in a dental anatomy course enhances the manual dexterity of D1 students, students need additional methods to enhance their knowledge, dexterity, and their clinical skills.

In 2020, a “Teeth Drawing Module” was implemented within the D1 dental anatomy curriculum to enhance students' understanding of tooth morphology, their dexterity, and their clinical skills compared with students who completed the traditional waxing dental anatomy course. In this new drawing module, they learned to draw accurate outlines and morphology of teeth. At the end of the module, students' feedback, perception, and preferences were assessed with a comprehensive survey. Students' grades in the drawing module, their waxing skills assessments, and their didactic exams were used to evaluate and assess the correlation between their drawing aptitude and their manual skills.

The aim of this intervention study was to (1) determine whether students who participated in the dental anatomy course that included the drawing module had better didactic and clinical dental anatomy skills than students who participated in the traditional dental anatomy course and (2) to evaluate students' perceptions of the tooth drawing module.

## 2. Materials and Methods

### 2.1. Description of the Course

The Dental Anatomy course at East Carolina University School of Dental Medicine includes a didactic and clinical component. The didactic component encompasses learning: (1) dental anatomy terminology, (2) the morphological characteristics of permanent and deciduous teeth, (3) each tooth's position in the mouth, and (4) physiologic considerations of each tooth.

Historically, the course was taught via lectures, interactive turning point exercises, handouts, and reading material based on the textbook “Concise Dental Anatomy and Morphology.” Students further learned the anatomical forms of teeth by analyzing dentoforms, large models of teeth, extracted human permanent teeth, and photographs shown on the computer screen. Didactic knowledge was assessed using computer-facilitated quizzes. The process assessment component included the wax build-up of various teeth.

At the end of the course, it was expected that students would be able to demonstrate knowledge of dental anatomy, terminology, and developmental chronology. Students were expected to demonstrate a mastery of their psychomotor skills by using the wax addition technique proficiently to reproduce natural tooth contours, and they demonstrated their cognitive skills and self-directed learning by critically self-evaluating the quality of their work.

### 2.2. Didactic Assessment

Ten computer-facilitated quizzes and one tooth identification examination were used to assess the students didactically.

#### 2.2.1. Computer-Facilitated Quizzes

Testing based on independent study from lectures and textbook assignments.

#### 2.2.2. Tooth Identification Exam

This exam was a computer-facilitated OSCE exam that presented teeth at stations for identification by tooth number. Cumulatively, the student identified 52 permanent human teeth when all stations were completed.

### 2.3. Waxing Assessment

Students completed daily exercises where they waxed multiple full crowns. Students received formative assessments after each 4 hr session. Once a student satisfactorily completed a formative assessment, she was allowed to sit for the corresponding summative skills assessment.

#### 2.3.1. Teeth Drawing Module

In 2020, the “Teeth Drawing Module” was implemented into the D1 dental anatomy course. In this module, first-year dental students learn to draw accurate representations of teeth which includes drawing the crown and root with all the featured anatomy in each aspect of a tooth. To learn to draw each tooth, students receive a manual drawing book that includes sample illustrations and instructions regarding how to draw the teeth, PowerPoint presentations, and illustration videos.

**Figure 1:**
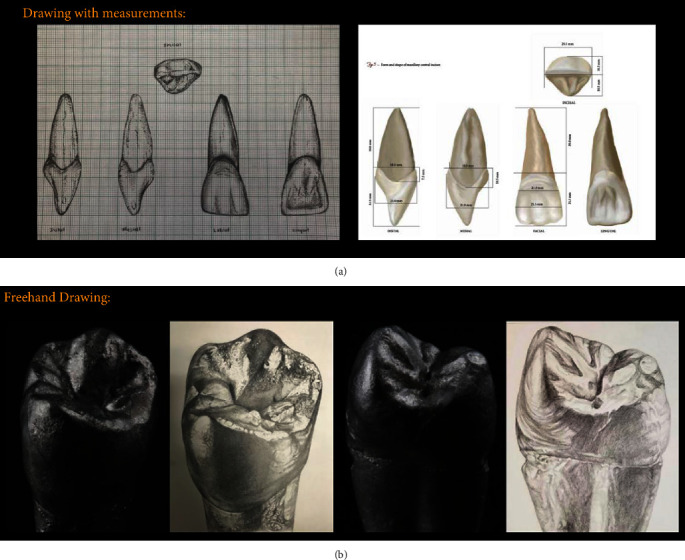
(a) A student's drawing of the five aspects of the maxillary central incisor on graph paper scaled by millimeter. Each square represents 1 mm in tooth measurements. (b) The student's artwork (white background) when compared with the original silver coated teeth pictures (black background) that students used on a sketch papers.

The students are required to complete two drawing projects:

(1) *Drawing with Measurements*. Photographs of the five aspects of each tooth (mesial, distal, labial or buccal, lingual, and incisal or occlusal) are superimposed on squared millimeter cross-section paper (Figure 1(a)).

Close observation of the outlines of the squared backgrounds shows the relationship of the crown to root, the extent of curvatures at various points, the inclination of roots, relative widths of occlusal surfaces, height of marginal ridges, contact areas, and so on. Additionally, students used a table of teeth measurements from Wheeler's Dental Anatomy, Physiology, and Occlusion book.

(2) *Freehand Drawing*. In this exercise, silver-coated teeth pictures were used to train students on how to see the light reflections, shadows, and contours and make sure how to replicate and draw the same picture. These basic skills were adapted to the situation of a tooth drawing (frame, contour, elements, shadows and highlights, composition) by Magne [5] when he applied that to the concept of the transition of his esthetics module and staging the students from 2D into 3D (Figure 1(b)).

#### 2.3.2. Teeth Drawing Exercise Assessment

**Figure 2:**
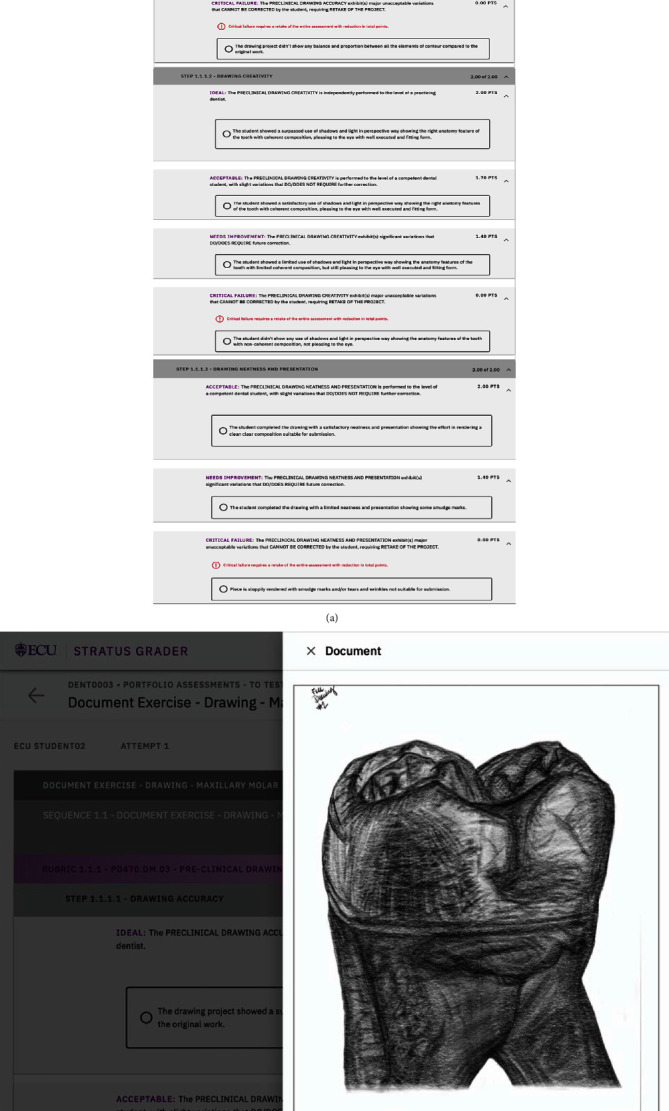
(a) The teeth drawing rubric that students and faculty used to evaluate students' teeth-drawing work. (b) A PDF file uploaded on XComP by a student.

A rubric was developed to assess performance. Three parameters were included in the rubric: (1) qualities and accuracy, (2) creativity, shadow, and light, and (3) neatness and presentation. Each parameter had three strata: acceptable, needs improvement, and critical failure (Figure 2(a)). Using this rubric, students self-assessed their drawings after submitting their artwork digitally (in .pdf format, Figure 2(b)) in XComP™, which is an online grading platform that allows faculty to upload rubrics and grade student work. XComP™ also allows students to submit their work digitally and self-assess their performance. Students had access to the rubric when self-assessing their artwork and were able to refer to the criteria and standards included in the rubric to guide their self-assessment. The faculty graders used the same rubric when evaluating the students' work. Faculty were blinded regarding which student submitted each assignment when completing the evaluations and providing feedback. A comparison between the faculty grade and the student self-assessment was made by XComP. All grades were reported on a grade card with any comments. Note that the goal of the self-assessment was to have the student make a realistic evaluation of their work as compared with the standard. The self-assessment has a numerical impact on the final grade.

### 2.4. Study Design

This intervention study began in 2019 when the drawing exercises were first offered in the dental anatomy course to the D1 Class of 2023. Both the Classes of 2023 (*n* = 51) and 2024 (*n* = 52) completed the drawing exercises. Those two classes were grouped together and considered as the test group. Students' grades in the drawing module, their waxing skills assessments, and their didactic exam grades were used to evaluate and assess the correlation between their drawing aptitude and their performance in the dental anatomy course. The Classes of 2021 (*n* = 51) and 2022 (*n* = 52) were grouped together and considered as the control group since these two classes did not have any drawing exercises. Students who had taken the course were compared with students who had not taken the course to determine if the drawing exercises improved students' understanding of tooth morphology, hand dexterity, and clinical skills.

A comprehensive survey was developed and distributed to the Classes of 2023 and 2024 immediately after they completed the dental anatomy course. The survey was distributed online using Qualtrics Survey software. Students had 2 weeks to finish the survey. IRB approval was obtained to distribute the survey (IRB #UMCIRB 19-002490). Completing the survey indicated the student's consent to participate in the study. The survey was specifically developed for the purpose of this study since other studies had not been conducted assessing students' opinions regarding the implementation of drawing exercises in dental anatomy courses. The Classes of 2021 and the 2022 did not receive the survey because the drawing exercises had not been included in the dental anatomy course when they took the course.

#### 2.4.1. Survey

The first section of the survey was developed to collect students' demographic data, student background, and their experience with art classes or other hands-on activities prior to commencing dental school. The second part of the survey evaluated: (1) students' perceived educational value of the drawing exercises; (2) students' perceptions regarding whether the drawing exercises correlated with their manual skills; (3) their preference regarding which drawing exercise they preferred (i.e., the measurements drawing or the freehand technique); (4) self-rating their waxing skills and drawing skills. The last part of the survey was designed to collect their feedback about incorporating this exercise in the school with future students. The full survey can be found in the Table S1.

### 2.5. Statistical Analysis

The survey data were collected from the Qualtrics Survey software using Microsoft Excel. Descriptive and bivariate analyses were conducted using SPSS 21.0 software (IBM Corp., Armonk, NY, USA). Frequency data were produced pertaining to the survey responses and students' final grades in the anatomy class pertaining to drawing scores, waxing scores, their ability to identify teeth, and their didactic content. Pearson correlations were calculated to assess whether significant correlations existed between final (i.e., end of the course) drawing scores and final: (1) waxing scores, (2) the identification of teeth, and (3) didactic scores. Independent *t*-tests were used to determine whether statistically significant differences existed between the mean waxing, tooth identification, and didactic final scores of students who completed the dental anatomy course prior to the introduction of the drawing exercises (Classes of 2021 and 2022) compared with students who completed the dental anatomy course that included the drawing exercise (Classes of 2023 and 2024). Statistical significance was set as *p* = 0.05. Answers to the last open-ended question were reviewed.

## 3. Results

### 3.1. Demographic Data

Demographic data of the control group (Classes of 2021 and 2022) and the test group (Classes of 2023 and 2024) are presented in Table 1.

There were 52 students in Class of 2023 and 52 students in Class of 2024. The overall response rate to the survey was 76%. Demographic data of students who completed the survey are presented in Table 2.

Tables 3 and 4 show the mean final scores and standard deviations of the waxing exercises, tooth ID exam, and didactic score among different classes. These scores were cumulative scores assessing multiple waxing exercises, multiple didactic assessments, and one tooth ID exam.

Classes of 2021 and 2022 were considered as the control group in which these two classes did not participate or had any drawing exercises; however, Classes of 2023 and 2024 had drawing exercises. It shows classes that had drawing exercises scored significantly higher in all other exercises compared with classes that did not have drawing exercises. As shown in Table 5, there is a significant positive correlation between drawing and waxing scores. There was a significant positive correlation between drawing and didactic scores.

**Figure 3:**
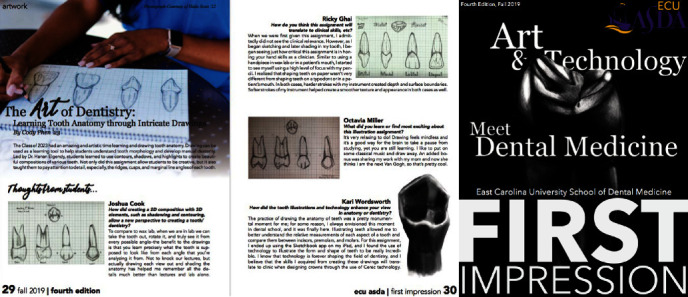
Some students' feedback that was published on the First Impression journal that ECU ASDA students shared with other students in the United States.

Tables 6 and 7 show the distribution of answers to quantitative questions in the survey.  Figure 3(a) shows selected students' comments on the drawing exercises. The survey results showed overall positive feedback. Most of the students found the drawing exercise enjoyable, relaxing, and was “break-time” from studying. Many students found that the drawing exercises supplemented the waxing portion of the course and enhanced their ability to learn the anatomy of the teeth. Overall, they enjoyed the course and hoped it would be implemented in the curriculum of future classes. Some students complained about the overload of exercises in the D1 courses, and they preferred to have fewer assignments while others preferred adding more drawing exercises.

## 4. Discussion

The purpose of this study was to assess whether incorporating a drawing module in a dental anatomy class improved students' final scores pertaining to waxing, identifying teeth, and didactic coursework. This study found that compared with students in the preceding 2 years, students who completed the drawing module were more likely to score higher in these areas. Furthermore, students' drawing scores were associated with how they did within the dental anatomy class pertaining to waxing exercises, tooth identification, and didactic exams.

The ability to draw an accurate outline of a tooth is a good indication that a student has clearly seen and understood its external morphology. Along with the visual component, this exercise helps the student develop the manual skills needed to correctly reproduce the anatomical form of teeth, which is crucial in almost any phase of dentistry [6].

When it comes to dental education, dental students are the main stakeholders of the educational process. Taking their perspective and their feedback about the evolving techniques is so crucial to enhance their educational learning experience [6].

Because dentistry is a school in art and science, incorporating art in the early stages of their education will enhance their creativity and their right side of their brain which is responsible for creativity, imagination, and art [7]. The right side of the brain is more visual and deals in images more than words. It processes information in an intuitive and simultaneous manner. It takes in the big picture and then looks at the details.

Dental school is a demanding environment that involves always blending science and art while developing, mastering, and demonstrating skills in a variety of areas including manual dexterity [8]. Understanding the significance of manual dexterity in dental careers will help students reconfirm if dentistry is the right fit.

The American Dental Education Association (ADEA) clearly states that it is important to fine tune manual dexterity skills before applying to dental school, especially when the Dental Admission Test (DAT) contains a section that specifically tests this skill, and during on-campus interviews, most dental school admissions staff will ask to discuss how you have developed your manual dexterity skills.

The ADEA wibsite lists drawing, painting, soap carving, and creating 3D artwork, as their top list of activities to fine-tune motor skills. Imam [9] also recommends drawing as an important tool for improving the manual dexterity of dental students.

Al-Johany et al. [10] found that there is a strong link between writing ability and drawing skills with dental skills and such components should be assessed by the examination system to identify the best candidates; however, these studies were used to predict manual dexterity before applying to dental school.

Many studies have attempted to determine the predictors of manual skills among preclinical students. A previous study showed that there may be a positive relationship between preclinical and clinical performance of these students when using dental students' preclinical performance as an indicator of clinical success [11].

Gillet et al. [12] investigated whether it is possible to predict the manual aptitude of a dental student through tests that allow the qualities of reflection and organization to be judged. They administered writing tests and drawing tests to 45 students. Another study by Walcott et al. [13] used waxing tests as predictors of students' performance in preclinical dentistry. These waxing tests may be useful in the early identification of students with psychomotor deficiencies for admission decisions and/or for designing and evaluating the effectiveness of early instructional intervention strategies on performance.

Another study by Park et al. [14] was to predict dental school performance based on prior dental experience and exposure to whether the variables of students with prior dental assisting experience and students with a parent who is a dentist can be used as predictors of students' preclinical and clinical course performance in dental school.

The present study showed classes which had drawing exercises scored significantly higher in all other exercises compared with classes which did not have drawing exercises. There was a significant positive correlation between drawing and waxing scores and the didactic scores.

This study had a great impact on improving students' skills and knowledge when compared to the two classes that did not have the drawing exercises. A total of 63% of the students showed that the drawing exercises made them like the dental anatomy course more, 14% showed that the drawing exercises had no impact on the course, and only around 5% showed that the drawing exercises made them like the course less.

The students had varied opinions regarding each drawing exercise. More than 37% of the class found both drawing assignments helpful, while 38% of students preferred the drawing with measurement. There were a lot of varied comments regarding what they preferred each exercise. Some respondents found that freehand drawing helped the most because it forced them to pay more attention to details whereas the measured drawings consisted more of counting boxes and replicating the existing picture. In Contrast, other respondents found that they were more concentrated on details when they completed the measurement drawings.

## 5. Conclusion

Teaching dental students drawing exercises has yielded positive outcomes, improving their manual dexterity, spatial understanding of anatomical information, and knowledge of dental anatomy. To optimize the effectiveness of drawing exercises, future studies should investigate the ideal frequency of these exercises in the course curriculum. Furthermore, evaluating students' clinical skills as they progress through their dental education can determine whether participating in drawing exercises affects their performance compared with nonparticipating peers.

## Figures and Tables

**Table 1:** Demographic information of each class (total number of students, gender, and age).

	Class of 2021	Class of 2022	Class of 2023	Class of 2024
Number of students	52	52	52	52
Gender				
Male	23	23	26	23
Female	29	29	26	29
Age				
Average	25	23	24	23
Youngest	21	19	20	20
Oldest	38	32	42	38

**Table 2:** Demographic information of students who completed the survey (gender/predental education level).

Background of dental students	*N* (%)
Gender	
Male	36 (51.9)
Female	41 (45.6)
No answer	2 (2.5)
Hands-on art classes experience	
No experience	36 (45.6)
Took some classes or majored in art	20 (25.3)
Took art classes as a hobby	2 (2.5)
More than one type of experience	16 (20.3)
Other/no answer	5 (6.3)
Other hands-on activities experience	
No experience	21 (26.6)
Professional dental work experience (dental assistant, hygienist, dental lab technician, etc.)	12 (15.2)
Hobby (woodworking, jewelry making, plays an instrument, sewing, etc.)	26 (32.9)
Both	20 (25.3)

**Table 3:** Mean scores of each class.

Exercise	Mean (±SD)			
	Class of 2021	Class of 2022	Class of 2023	Class of 2024
Waxing	61.56 (±18.65)	63.71 (±21.47)	84.58 (±6.24)	79.89 (±8.79)
Tooth ID	70.74 (±10.94)	63.24 (±9.47)	86.58 (±8.81)	85.54 (±7.67)
Didactic	83.84 (±8.53)	84.14 (±6.62)	90.25 (±5.42)	89.71 (±4.68)

**Table 4:** Comparison of mean scores between classes that had drawing exercisers vs. classes that did not have drawing exercisers.

Exercise	Mean (±SD)		*t*-test	*df*	*p*-value
	Classes of 2021 and 2022 (no drawing exercises) (*n* = 103)	Classes of 2023 and 2024 (had drawing exercises) (*n* = 103)			
Waxing	62.65 (±20.06)	82.21 (±7.95)	−9.202	133.316	0.001^*∗∗*^
Tooth ID	66.95 (±10.85)	86.06 (±8.23)	−14.240	190.176	0.001^*∗∗*^
Didactic	83.99 (±7.59)	89.98 (±5.04)	−6.663	177.364	0.001^*∗∗*^

^*∗∗*^*p* < 0.01.

**Table 5:** Pearson correlation between drawing exercises scores and other exams, Classes of 2023 and 2024 (*N* = 103).

	Mean (±SD)	Pearson's *r*	*p*-value
Drawing (%)	93.59 (±7.71)		
Waxing (%)	82.21 (±7.95)	0.244	0.013^*∗*^
Tooth ID (%)	86.06 (±8.23)	0.066	0.510
Didactic (%)	89.98 (±5.04)	0.341	0.001^*∗∗*^

^*∗*^*p* < 0.05 (correlation is significant at the 0.05 level).  ^*∗∗*^*p* < 0.01 (correlation is significant at the 0.01 level).

**Table 6:** Distribution of answers to the first 14 questions in the survey.

Question	Distribution (%) (students did not answer the questions were excluded)				Numbers of students who did not answer the question
	Agree	Neutral	Disagree	Do not know

Q1: Before starting the drawing exercises, I thought I would be able to pass the exercises.	54 (68.4)	9 (11.4)	13 (16.5)	1 (1.3)	2
Q2: Participating in the drawing exercises improved my waxing skills.	50 (63.3)	12 (15.2)	13 (16.5)	2 (2.5)	2
Q3: The drawing exercise helped me to better understand the anatomy of the teeth.	64 (81.0)	4 (5.1)	8 (10.1)	0 (0)	3
Q4: The drawing exercise helped me to better understand the occlusion course.	34 (43.0)	20 (25.3)	20 (25.3)	0 (0)	5
Q5: The drawing exercises helped me incorporate dental anatomy more effectively into the waxing exercises.	55 (69.6)	12 (15.2)	10 (12.7)	0 (0)	2
Q6: I understood how the drawing exercise was correlated to the waxing exercise.	62 (78.5)	7 (8.9)	7 (8.9)	0 (0)	3
Q7: I developed better fine motor skills by completing the drawing exercises.	52 (65.8)	11 (13.9)	11 (13.9)	3 (3.8)	2

	Likely	A little/somewhat unlikely	Very unlikely	Do not know	

Q8: My ability to visualize tooth anatomy details improved because of the drawing exercises.	63 (79.7)	10 (12.7)	3 (3.8)	1 (1.3)	2
Q9: How likely are you to recommend this drawing exercises to friends or colleagues?	58 (73.4)	11 (13.9)	7 (8.9)	1 (1.3)	2
Q10: The drawing exercises should be continued with future students.	67 (84.8)	3 (3.8)	4 (5.1)	3 (3.8)	2

	Drawing with measurements	Freehand drawings	Both of them	None of them	

Q11: During the course, you experienced two different types of the drawing assignments, which one you see was more helpful.	29 (36.7)	14 (17.7)	28 (35.4)	5 (6.3)	3

	It made me like the course more	Natural	It made me like the course less	It had no impact	

Q12: How did the drawing exercises impact your perception of the dental anatomy course?	51 (64.6)	13 (16.5)	4 (5.1)	9 (11.4)	6

	Excellent/very good	Good	Fair	Poor	

Q13: How would you rate your skills on the drawing exercises?	42 (53.2)	17 (21.5)	15 (19.0)	3 (3.8)	2
Q14: How would you rate your skills on the waxing exercises?	67 (84.8)	9 (11.4)	1 (1.3)	0 (0)	2

**Table 7:** Overall satisfaction score of the drawing exercise.

Q15: Overall satisfaction level^a^	Very satisfied	Somewhat satisfied	Satisfied	Neutral	Dissatisfied	Somewhat dissatisfied	Very dissatisfied
Overall, how satisfied or dissatisfied are you with your experience with the drawing exercises?	38 (48.1)	18 (22.8)	10 (12.7)	8 (10.1)	None	None	3 (3.8)

^a^Two students did not answer this question.

## Data Availability

Tables' and figures' data used to support the findings of this study are included within the article and are available from the corresponding author upon request.

## Additional Points

*Limitations of the Study*. Our study did not show causality, only associations. Findings from the study implied that incorporating drawing into the course may improve other scores in the class; however, since we did not assess the drawing skills of the Classes of 2021 and 2022, we do not know if the Classes of 2023 and 2024 inherently had better hand skills than the previous classes.

## Supplementary Materials

Supplementary Materials Table S1: Teeth drawing exercise survey.
