# Supplementary material for: Teaching Dental Drawings for Freshman Dental Students and Its Correlation with Manual Dexterity
Source: Int J Dent. 2023 Jun 1;2023:5685003. doi: 10.1155/2023/5685003 (PMC10250097; doi:10.1155/2023/5685003)
Supplement: Supplementary Materials — Table S1: Teeth drawing exercise survey. [file 5685003.f1.docx]

**Supplementary material**

Teeth Drawing Exercise Survey

| Question | Scale | Category |
| --- | --- | --- |
| a. Please indicate whether you have experienced hands-on art classes in the following settings: | 0=No  1=Took some kind of classes  2=Hobby  3=Other / No answer  4=Choose more than one answer | background |
| b. Please indicate your experience with other hands-on activities: | 0=No  1=Professional dental work  2=Hobby  3= Both professional dental work and hobby | background |
| c. What is your gender? | Female=1. Male=2 | background |
| Q1 Before starting the drawing exercises, I thought I would be able to pass the exercises. | 3= Agree  2= Neutral  1= Disagree  0= Do not know or NA | preferences |
| Q2 Participating in the drawing exercises improved my waxing skills. | 3= Agree  2= Neutral  1= Disagree  0= Do not know or NA | skills |
| Q3 The drawing exercise helped me to better understand the anatomy of the teeth. | 3= Agree  2= Neutral  1= Disagree  0= Do not know or NA | didactic |
| Q4 The drawing exercise helped me to better understand the occlusion course. | 3= Agree  2= Neutral  1= Disagree  0= Do not know or NA | didactic |
| Q5 The drawing exercises helped me incorporate dental anatomy more effectively into the waxing exercises. | 3= Agree  2= Neutral  1= Disagree  0= Do not know or NA | skills |
| Q6 I understood how the drawing exercise was correlated to the waxing exercise. | 3= Agree  2= Neutral  1= Disagree  0= Do not know or NA | Incorporation and correlation |
| Q7 I developed better fine motor skills by completing the drawing exercises. | 3= Agree  2= Neutral  1= Disagree  0= Do not know or NA | skills |
| Q8 My ability to visualize tooth anatomy details improved because of the drawing exercises. | 3= Likely  2= A little / somewhat unlikely  1= Very unlikely  0= Do not know or NA | skills |
| Q9 How likely are you to recommend this drawing exercises to friends or colleagues? | 3= Likely  2= A little / somewhat unlikely  1= Very unlikely  0= Do not know or NA | Experience |
| Q10 The drawing exercises should be continued with future students. | 3= Likely  2= A little / somewhat unlikely  1= Very unlikely  0= Do not know or NA | Experience |
| Q11 During the course, you experienced two different types of the drawing assignments, which one you see was more helpful. | 1=Drawing with measurements  2=Free-hand drawings  3=Both of them  0=None of them | preferences |
| Q12 How did the drawing exercises impact your perception of the dental anatomy course? | 0=It had no impact  1=It made me like the course less  2=Natural  3= It made me like the course more | Incorporation and correlation |
| Q13 How would you rate your skills on the drawing exercises? | 3= Excellent / very good  2= Good  1= Fair  0= Poor | skills |
| Q14 How would you rate your skills on the waxing exercises? | 3= Excellent / very good  2= Good  1= Fair  0= Poor | skills |
| Q15 Overall, how satisfied or dissatisfied are you with your experience with the drawing exercises? | 7=Very Satisfied  6=Somewhat Satisfied  5=Satisfied  4=Neutral  3=Dissatisfied  2=Somewhat Dissatisfied  1=Very Dissatisfied | Overall |
